# Diagnostic Methods of Common Intestinal Protozoa: Current and Future Immunological and Molecular Methods

**DOI:** 10.3390/tropicalmed7100253

**Published:** 2022-09-21

**Authors:** Loeki Enggar Fitri, Didi Candradikusuma, Yulia Dwi Setia, Purwa Adrianta Wibawa, Agustin Iskandar, Nuning Winaris, Aulia Rahmi Pawestri

**Affiliations:** 1 Department of Parasitology, Faculty of Medicine, Universitas Brawijaya, Malang 65145, Indonesia; 2 Department of Internal Medicine, Faculty of Medicine, Universitas Brawijaya, Malang 65145, Indonesia; 3 Department of Clinical Pathology, Faculty of Medicine, Universitas Brawijaya, Malang 65145, Indonesia

**Keywords:** *Entamoeba histolytica*, *Giardia* spp., *Cryptosporidium* spp., immunodiagnosis, molecular diagnosis

## Abstract

Intestinal protozoan infection is a persisting public health problem affecting the populations of developing countries in the tropical and subtropical regions. The diagnosis of intestinal protozoa remains a challenge especially in developing countries due to a shortage of laboratory facilities, limited health funding, and the remoteness of communities. Despite still being widely used, conventional diagnoses using microscopy and staining methods pose important limitations, particularly due to their low sensitivities and specificities. The selection of diagnostic methods needs to be carefully considered based on the objective of examination, availability of resources, and the expected parasite to be found. In this review, we describe various immunodiagnosis and molecular diagnostic methods for intestinal protozoa infection, including their advantages, disadvantages, and suitability for different settings, with a focus on *Entamoeba histolytica*, *Giardia duodenalis*, and *Cryptosporidium* spp.

## 1. Introduction

Parasitic infections affect millions of people worldwide and result in significant morbidities and mortalities, especially in low- and middle-income countries. According to the World Health Organization, intestinal parasitic infections affect approximately 67.2 million illnesses, equivalent to 492,000 disability-adjusted life years/DALYs [1]. Intestinal protozoa, common intestinal infectious agents, are phylogenetically diverse and are broadly distributed across human and animal populations. Although often associated with asymptomatic infection, they can contribute to important pathologies. Host–parasites interactions can range from commensalism to parasitism, and some are even suggested to have evolved into mutualistic associations [2].

Intestinal protozoa infections are an important cause of morbidity and mortality, especially with the emergence of immunosuppressive diseases. The wide spread of the human immunodeficiency virus (HIV)/acquired immunodeficiency syndrome (AIDS) in the world shifted the nature of intestinal protozoan infection. Those parasites that normally cause sporadic or zoonotic diseases with benign or asymptomatic infections have become opportunistic, causing severe episodes of diarrhea in patients with HIV/AIDS. Understanding the diversity of these eukaryotes in the gut of healthy individuals and immunocompromised patients is necessary for understanding their role in both health and disease and when considering the sensitive tests used to diagnose intestinal protozoa infections in these immunocompromised patients [3].

Microscopic examination is the traditional method for fecal parasite diagnosis. Although using the microscopic tool is laborious and requires experienced examiners for optimal interpretation, this method is still widely used for the diagnosis of intestinal protozoa, especially in areas with limited settings. However, many laboratories lack skilled examiners who are capable of reliably identifying the presence and type of intestinal protozoa. This leads to the inability to accurately identify protozoa, differentiate pathogenic from non-pathogenic species, and discriminate artefacts on microscopic examinations, resulting in limited sensitivity and specificity of intestinal protozoa diagnosis [4].

The development of commercial and in-house immunodiagnostic methods, either to detect parasite antigens or host antibodies, increases the sensitivity and specificity of the detection of protozoan infection. Refinement and broader use of nucleic-acid-based detection techniques with high sensitivities and specificities also allow accurate detection of intestinal protozoa. Moreover, they enable the determination of molecular epidemiology, host range, and possible transmission routes of intestinal protozoa. Although the selection of diagnostic methods heavily depends on the objectives, expected parasite to be found, and resource availability, new diagnostic technologies will generate more comprehensive knowledge on intestinal protozoan diversity and their link with health and disease [5]. 

Here, we review numerous relevant studies covering intestinal protozoa diagnosis. Three intestinal protozoa, *Entamoeba histolytica*, *Giardia duodenalis,* and *Cryptosporidium* spp., are prioritized since they have been reported to cause significant disease burdens, and the number of citations for these parasites is sharply rising [1]. This review discusses recent immunological and molecular diagnostic approaches for these common intestinal protozoa. Although the definitive diagnosis of these parasites remains relatively difficult using the conventional method, the proper selection of immunodiagnostic and molecular diagnostic methods can greatly improve the reliability of detection, leading to proper management for the patients and effective control strategies for the community.

## 2. Challenges in Identification of Human Intestinal Eukaryotic Diversity 

Intestinal protozoa, such as *Entamoeba histolytica*, are the major causes of diarrhea in adults, while Cryptosporidium spp. and *Giardia duodenalis* (synonymous with Giardia lamblia) often affect children. Other relatively common intestinal protozoa are *Blastocystis* spp. and *Dientemoeba fragilis*. The latter two species are found with varying prevalence among populations [6], although they are occasionally identified as commensal parasites. 

*Entamoeba* spp., including *E. histolytica*, demonstrates a high level of prevalence among humans, chimpanzees, and baboons in ecosystems where the human and nonhuman primate populations co-exist (~60% for all *Entamoeba* spp. and ~10% of *E. histolytica*), highlighting the potential for the zoonotic transmission of *Entamoeba* species. Notably, in contrast to human infections, the presence of *E. histolytica* in chimpanzees was never associated with symptoms in a tested population [7]. In cases of amebiasis, the microscopic method could not differentiate *E. histolytica* from closely related species, such as *E. dispar* and *E. moshkovskii*. Without the evidence of erythrophagocytosis, *E. histolytica* is indistinguishable from *E. dispar* and should, therefore, be noted as *E. histolytica/dispar* [2].

Similarly, giardiasis, which is brought on by *G. duodenalis*, can result in asymptomatic infection or abdominal pain, persistent diarrhea, and vomiting, which have a major negative impact on human health. Cattle are one of the main hosts of *G. duodenalis*, which can excrete cysts which are a potential threat to humans. A survey for *G. duodenalis* among cattle in Scotland further illustrated the importance of surveillance in animal populations, since this species was shown to be commonly found across tested beef and dairy cattle (~32%), which included genetic assemblages associated with human symptomatic infections [8]. Meanwhile, the detection of *G. duodenalis* via a permanent stained smear with chlorazol black dye, which has a higher standard than trichrome, only provided a sensitivity of 66.4% [2]. 

The majority of reported cases of human cryptosporidiosis are caused by several *Cryptosporidium* species, including *C. hominis*, *C. parvum*, *C. meleagridis*, *C. felis*, *C. canis*, *C. cuniculus*, and *C. ubiquitum*, with *C. parvum* and *C. hominis* being the most prevalent disease-causing species in humans [9]. *Cryptosporidium* *hominis* is the species which infects only humans, while *C. parvum* infects humans and cattle [5]. Like all coccidian intestinal parasites, small and poorly stained *Cryptosporidium* spp. oocysts can be easily missed in routine microscopic examination. The sensitivity of light microscopy is increased by performing modified acid-fast stain, although this modification has been shown to be associated with a sensitivity of only 54.8%. Furthermore, modified acid-fast stain staining is usually only performed at special request or if the microscopist detects structures suspicious for *Cryptosporidium* spp. on permanent stains. Under modified acid-fast stain staining, the oocyst of *Cryptosporidium* spp. appears as a bright-red sphere containing four crescent-shaped sporozoites, although, occasionally, oocysts can also appear as transparent “ghost” cells [2]. 

There are several contradictory datasets regarding the potential role of *Blastocystis* spp. and *D. fragilis* in both disease and health. Whilst potentially misleading detection tools can explain some variation between studies, this controversy might also result from the vast genetic diversity within *Blastocystis* spp. At least seven morphologically identical but genetically different organisms make up the species *Blastocystis hominis* [4,6,10]. Meanwhile, there have been reports that certain subtypes of *D. fragilis* exhibit unique virulence traits, including both pathogenic and non-pathogenic variations [10].

## 3. The Development of Immunological Methods

Immunodiagnostic tests are generally inexpensive, user-friendly, and enable fast-obtained results. Antibody and antigen detection tests, such as indirect hemagglutination (IHA), indirect immunofluorescence (IIF), enzyme-linked immunosorbent assay (ELISA), direct fluorescent antibody (DFA), rapid enzyme immune assay (EIA), immunochromatographic test (ICT), or latex agglutination, are commercially available via several different platforms [11]. The combination of antibody and fecal antigen detection assays is more sensitive and specific than microscopy for the diagnosis of several intestinal protozoan infections [12].

### 3.1. Immunodiagnostic Methods for Amoebiasis

For the diagnosis of *E. histolytica* infections, IHA and IIF methods can be performed. However, one of the most popular and widely used platforms is the ELISA. Variations in this platform can be used to detect either antigens during intestinal amoebiasis or anti-*Entamoeba* antibodies during amoebic liver abscess (ALA) [13], with several ELISA kits being commercially available. In addition to fecal specimens, serum or liver abscess aspirates can also be subjected to this method [14,15].

Several studies developed monoclonal antibody-based platforms using various *E. histolytica* antigens, such as lectin-rich surface antigen, lipophosphoglycan [16], and the 170-kDa amoebic adherence lectin [17]. In areas of the world where *E. histolytica* infection is endemic or if intestinal amoebiasis is specifically suspected by a physician, antigen-based tests can be performed. These tests usually employ monoclonal antibodies against the *E. histolytica* adhesin Gal/GalNAc lectin. This antigen has also been reported to be detectable in the sera and saliva of patients, although further evaluations are required [15]. 

Nevertheless, not all commercially available antigen tests can differentiate between *E. histolytica* and *E. dispar*. The sensitivity of the *E. histolytica* antigen detection tests ranges from 80% to 94% compared to that of polymerase chain reaction (PCR), with one study reporting a commercial, ELISA-based platform to be less sensitive than microscopy. Although antigen-detection tests proved to be useful for intestinal amoebiasis detection, they possess some crucial limitations, including the need for fresh or unpreserved fecal samples and the inability to differentiate *E. histolytica* from *E. dispar* and *E. moshkovskii* [18,19].

When evaluating patients from *E. histolytica*-endemic areas, it is important to notice that immunoassays detecting anti-*E. histolytica* antibodies turn negative earlier following the treatment of extraintestinal amoebiasis compared to IHA-based tests, which remain positive for at least 6 months following treatment [20]. An in-house point-of-care antibody-detecting test using a dipstick platform has also been developed with a claimed sensitivity of 98.1% compared to a commercial latex agglutination test [21]. Overall, antibody detection tests have shown good performance in the diagnosis of extraintestinal amoebiasis but are less practical for the detection of intestinal amoebiasis, patients in endemic areas with a high baseline antibody, and immunocompromised patients. 

A rapid IC assay to simultaneously detect *E. histolytica/E. dispar, G. duodenalis,* and *C. parvum* was also commercially available. This strip assay used monoclonal antibodies against proteins of these protozoa and claimed a nearly perfect sensitivity and specificity [22]. However, like several aforementioned immunodiagnostic platforms, it failed to distinguish between the pathogenic *E. histolytica* and the non-pathogenic *E. dispar* and has been discontinued from the market.

### 3.2. Immunodiagnostic Methods for Giardiasis

The detection of *Giardia duodenalis* has been enhanced using antigen detection methods. Some immunoassays for *Giardia* are commercially available and widely used in clinical laboratories. The ELISA platform for *Giardia duodenalis* that has been approved by the World Health Organization is a rapid, sensitive, specific, and inexpensive method of confirming *Giardia duodenalis* coproantigens even in the absence of live parasites in the fecal samples [23]. 

Several previous studies found that the commercial DFA test used to detect *Giardia duodenalis* showed a sensitivity of 96–99% and specificity of 100%. This test utilizes fluorescein-labeled antibodies directed against the cell wall proteins of *Giardia duodenalis* cysts and allows visualization of the intact parasites, thus, providing a definitive diagnosis with a greater sensitivity than the conventional permanent smears [4,24].

For laboratories with limited capacity for diagnostic complexity, simple EIAs and ICTs are commercially available for the detection of *Giardia duodenalis*. Rapid immunoassays based on immunochromatographic lateral flow for *Giardia duodenalis* have become a popular diagnostic tool because they do not require trained microscopists, expensive equipment and can be completed very quickly. Meanwhile EIA-based tests might be more appropriate for screening in high-prevalence areas [4]. A study comparing four EIAs found sensitivities ranging between 63% and 91% and specificities of 95%. Another study demonstrated 94% to 100% sensitivity and 100% specificity when five *Giardia duodenalis* EIAs were evaluated [4,24]. Although the aforementioned methods are able to detect the *Giardia duodenalis* species with prominent sensitivities and specificities, no immunological test to date can differentiate the *Giardia duodenalis* assemblages in clinical samples. 

### 3.3. Immunodiagnostic Methods for Cryptosporidiosis

Direct immunofluorescence microscopy, ELISA, and ICTs are three methods that have been successful in the immunological detection of *Cryptosporidium* spp. oocyst antigens, and a number of commercial kits are available. Compared to traditional stains, immunofluorescence kits are more sensitive and specific in detecting *Cryptosporidium* spp. oocysts in fecal smears. *Cryptosporidium* spp., using direct immunofluorescence from fecal samples using fluorescein isothiocyanate (FITC)-labelled monoclonal antibodies, works against surface-exposed epitopes of oocysts. It was reported to have nearly perfect sensitivity and specificities against this protozoan, although it could not distinguish different species of *Cryptosporidium* [24,25,26,27,28].

Several studies evaluated the sensitivities and specificities of the available kits for cryptosporidiosis and found overall similar performance levels for EIA- and DFA-based methods (90% sensitivity; 95% specificity) [4]. Some commercially available immunoassays allow simultaneous and rapid detection of *Giardia duodenalis* and *Cryptosporidium* spp. These tests, including EIA, ICT, and DFA assays, are favorable since coinfections of both protozoa are commonly found [24,25,26,27]. Since HIV-infected and immunocompromised individuals are particularly at risk for serious complications from these coccidian parasites, clinicians should consider routinely suggesting at least DFA and molecular testing, if available, for patients with suspected cryptosporidiosis [4].

An ELISA test is performed to detect the presence of soluble *Cryptosporidium* spp. coproantigens. Depending on the commercial kit, a combination of monoclonal and polyclonal antibodies is used to collect and identify *Cryptosporidium* spp. coproantigens. These tests were developed to identify antigens from *C. parvum* in fecal samples, but they can also identify common epitopes from infections with other *Cryptosporidium* species [28].

Rapid, ICT-based methods for cryptosporidiosis are significantly less sensitive, with a multi-institutional study reporting a sensitivity of between 50.1% and 86.7% depending on the test manufacturer [4]. However, a rapid immunochromatographic assay kit for the detection of both *Giardia duodenalis* and *Cryptosporidium* spp. is also available with superior specificities and sensitivities. This monoclonal, antibody-based platform is quick, easy, and simple to interpret [24,25,26,27].

## 4. The Establishment and Expansion of Molecular Methods 

### 4.1. Polymerase Chain Reaction (PCR)-Based Methods

Over the past decades, the development of molecular methods to diagnose intestinal parasite has been centered on PCR assays. Various PCR-based detection methods have been performed to investigate the presence of intestinal parasites, mainly in stool samples. Previously, simple-yet-sensitive PCR assays, such as random amplified polymorphic DNA (RAPD), PCR-restriction fragment length polymorphism (PCR-RFLP), amplification fragment length polymorphism (AFLP), single-strand conformation polymorphism (SSCP), and loop-mediated isothermal amplification (LAMP), were used to analyze the genetic variation and identify the genus, species, or strain level of parasites [29]. In addition, nested PCR, in which the amplicons from the first PCR reaction are used as a template for the second PCR reaction, was also used in previous studies to identify various intestinal protozoa obtained from stool samples [30,31].

#### 4.1.1. PCR for the Diagnosis of Amoebiasis

PCR assays targeting 18S rDNA are widely used for detection and identification of the *Entamoeba* species. This species can be easily detected in a single copy of a DNA fragment from a gene or multicopy, extrachromosomal plasmid in amoeba. The amplification of *E. histolytica* and *E. dispar* DNA fragments from human stool by conventional PCR has been established to be a sensitive and specific method for their detection. A PCR test for the identification of *E. moshkovskii* was developed, and it was shown to have a high sensitivity and specificity using DNA extracted directly from stool samples [16,18].

Multiplex assays and LAMP simplify the molecular detection of intestinal protozoa. Liang et al. reported the development of a LAMP procedure for *E. histolytica* detection. This one-step procedure claimed a detection threshold of one parasite per reaction with good specificities since it did not detect nucleic acid from other *Entamoeba* species, bacteria, or viruses [32].

#### 4.1.2. PCR for Giardiasis

PCR-based methods are often limited to research laboratories and are mainly used for subtyping, such as the determination of assemblies or subassemblies of *G. duodenalis*. Mayor, the target gene sequence used in several molecular studies on *Giardia* species, include the genes encoding the ribosomal small subunit RNA (SSU), glutamate dehydrogenase (gdh), triosphosphate isomerase (tpi), and β genes giardine (a protein in the adhesive disc of *Giardia duodenalis*). Comparison and polymorphisms of glutamate dehydrogenase (gdh), the small subunit of ribosomal RNA (SSU), and triose phosphate isomerase (tpi) genes showed that *G. duodenalis* can be classified into at least eight different genetic groups (from A to H). All these assemblies are indistinguishable by light microscopy. Two assemblies, A and B, are mainly isolated from humans [33].

#### 4.1.3. PCR for Cryptosporidiosis

PCR is claimed to be a powerful method with higher sensitivity and specificity than microscopy. In 1998, Morgan et al. performed a comparison study with more than 500 stool samples using both microscopy and PCR methods. The study aimed to detect *C. parvum*. The microscopic diagnosis was conducted with Ziehl–Neelsen stain, while conventional PCR was also performed for screening using primers designed to amplify the *C. parvum* 18S rRNA gene [34]. More importantly, PCR can be used to differentiate *Cryptosporidium* genotypes [35], thus, aiding in the molecular epidemiology of this protozoa. The identification of host-adapted subtype groups *C. parvum* and *C. hominis* was achieved by the use of sequence analysis of the 60-kDa glycoprotein (gp60) gene. Furthermore, for some other *Cryptosporidium* species, *including C. meleagridis, C. ubiquitum, C. viatorum, C. ryanae*, and *Cryptosporidium* chipmunk genotype I, a gp60-based subtyping tool has recently been established [9]. This information shows that PCR is a cost-effective high-throughput screening method compared to microscopy.

LAMP was also developed to detect *Cryptosporidium* spp. oocysts in stool and environmental samples [36]. Furthermore, LAMP was shown to be superior to nested PCR for the diagnosis of *Cryptosporidium* spp. in the veterinary field [37].

In addition, Luminex-based assays can also be used to differentiate *C. parvum* and *C. hominis*. The species-specific probes are conjugated to carboxylated Luminex microspheres and hybridize to the *Cryptosporidium* spp. microsatellite-2 region. This assay showed a specificity of 100% and was shown to be more sensitive than the direct fluorescent assay [38].

#### 4.1.4. Real-Time PCR

In recent years, real-time PCR has been widely used as a diagnostic tool for intestinal parasite infection, both in multiplex [39,40] and multi-parallel [41] approaches. Multi-parallel qPCR is considered to be more applicable in resource-limited settings compared to multiplex qPCR as a less sophisticated and more acquirable apparatus can be used [41,42].

A comparison study was performed by Hove et al. (2009) to establish a new diagnostic strategy for intestinal parasite detection among Europe’s returning travelers and immigrants. In this study, they performed multiplex real-time PCR, a fluorescence-based quantitative PCR using multiple design primers, which allowed the detection several intestinal protozoa, including *E. histolytica*, *G. duodenalis*, and *Cryptosporidium*. The comparison was made between weekly-basis PCR tests and daily microscopy, as well as coproantigen detections. The findings from the study concluded that multiplex real-time PCR significantly increased the detection rate of *E. histolytica*, *G. duodenalis*, and *Cryptosporidium*, therefore, establishing the supremacy of PCR over microscopy and antigen detection in this setting [43]. In addition, real-time PCR proved to be one of the most sensitive methods for intestinal parasite detection. A previous study compared the sensitivity and specificity of four diagnostic methods, namely formol-ethylacetate (FEA) concentration, salt–sugar flotation (SSF) concentration followed by microscopy of iodine-stained concentrate, immunofluorescence assay (IFA), and real-time polymerase chain reaction (qPCR) for detection *of G. duodenalis* in clinical fecal samples. The result showed that qPCR and IFA were significantly more sensitive than microscopy of iodine-stained concentrates using either FEA or SSF [25].

Primer design is one of the key points which determines the accuracy of a PCR test. Sensitivity and specificity of PCR detection depend on the chosen primers for the reaction. Primers to detect intestinal parasite are commonly designed based on the sequence of internal transcribed spacer (ITS1 or ITS2) and ribosomal RNA (18S rRNA) genes [41], which are known as highly conserved areas and can therefore be used to identify the taxonomy level of a microorganism. However, in some cases, the combination of multiple genes is needed to accurately distinguish certain species. A study performed by Weinreich et al. (2021) compared the sensitivity and specificity of primers designed based on the sequence of small subunit ribosomal RNA (SSU rRNA), the *Cryptosporidium* oocyst wall (COWP), and the DnaJ-like protein (DnaJ) genes in stool samples of Ghanaian HIV patients and military returnees. The result of the investigation showed that the SSU rRNA primers had the highest sensitivity in the PCR test, while the COWP primers had the highest specificity. They also suggested the use of the combination of primers from both genes for optimum accuracy of *Cryptosporidium* spp. detection in stool samples, particularly in low-prevalence settings [44].

Intestinal eukaryotic profiling is influenced by different methodologies at each step of the techniques. A previous study compared the efficacy of four DNA extraction protocols in recovering eukaryotic DNA from stool samples. The aforementioned study described significant differences in eukaryotic component recovery among the methods, for example, during the lysis and purification step [45].

### 4.2. Next-Generation Sequencing (NGS)

Nowadays, the development of molecular diagnostic has advanced towards metagenomics techniques. Next-generation sequencing (NGS) technologies have been broadly applied in various microbiome studies. Several attempts were made, using NGS to identify highly diverse parasites from various sources, such as shellfish [46], stool samples from horses [47], and surface, irrigation, and wastewater sources [48]. NGS is also known as a versatile technology for detecting mixed infection of parasites, as well as identifying a rare/novel subtype of parasitic infection. A study performed by Bailly et al. successfully detected mixed infection of *Cryptosporidium* species/subtypes from stool samples collected during the outbreak caused by contaminated drinking water supplies in Grasse in the south of France [49]. Similar studies used NGS to identify *Blastocystis* mixed-subtype infections [50] and *G. duodenalis* mixed-assemblage infections in cattle [51]. 

Unfortunately, it remains a challenge to apply NGS methods for the profiling of intestinal parasites, as the eukaryote genome is larger and more complex compared to the prokaryote genome. Furthermore, the prevalence of parasites is considerably low compared to that of bacteria in the environment and gastrointestinal tract. Additionally, some difficulties are expected while performing DNA extraction from (oo)cysts, and there is a limited parasite database availability for the development of a data analysis pipeline. Thus, the fundamental needs for NGS application include standardized protocols for the profiling of intestinal parasites in highly complex environments, such as stool samples, and well-established datasets for metagenomics analysis [52].

To study protozoan diversity in various samples, we can use both nontargeted (shotgun) and targeted metagenomics approaches. The nontargeted approach refers to a strategy where all microbial genomes present in a sample are fragmented and then sequenced on NGS platforms [53,54], while, in targeted approaches, taxon-specific primers are used to target conserved gene regions of protozoa [55,56]. Targeted metagenomics (amplicon-based metagenomics) often use conserved gene regions of only a single gene. However, with this approach, it is also possible to target multiple genes, which is known to increase the detection coverage of protozoa. ITS, 18S rRNA, or the large ribosomal subunit (LSU)/28S rRNA genes are often used as targets in metagenomics studies related to protozoa [53,57,58]. Commonly targeted regions are the V4, V5, and V9 of the 18S rRNA gene [48,59,60,61]. Among those regions, the V4 is known to have the longest sequence and the most conserved, variable regions; thus, it is considered to have the highest sensitivity for protozoan detection [54,62,63]. Many studies have successfully reported the detection of sequences of protozoa such as *E. histolytica*, *G. duodenalis*, *Cryptosporidium* spp., and *Blastocystis* spp. from various samples using the V4 regions of the 18S rRNA gene [48,59,61,64]. This emphasizes the importance of choosing variable regions of gene(s) as targets in metagenomics studies, which is also applicable for PCR methods.

NGS platforms used for metagenomic profiling also determine the diversity of protozoan parasites found in a sample. NGS adopts various platforms/systems, including Roche 454, Pacific Biosciences, Ion Torrent, Illumina/Solexa, and Oxford Nanopore [65]. The workflow of library preparation, sequencing, and raw data output are similar for all platforms [66]. The only difference among those platforms is the sequencing methods, which are known as pyrosequencing, sequencing by synthesis, sequencing by ligation, and ion semiconductor sequencing. Illumina (HiSeq and MISeq), one of the most commonly used platforms, uses the method called sequencing by synthesis. This sequencing method produces short read lengths which are best applied for species with smaller genomes, such as prokaryotes, and also to read short genome regions [67,68], for example, the regions of the 18S rRNA gene of protozoan parasites [48,60,61,69,70]. On the other hand, PacBio and Nanopore platforms can read longer sequences, which are easier for bioinformatics analysis. Therefore, those platforms are more advantageous for reading larger genome sequences, such as those of eukaryotes, including protozoan parasites. The selection of the appropriate NGS platform for sequencing depends on several factors, including the choice of metagenomics approach, research questions, costs, and the length of the genome [67,68].

The composition of the intestinal eukaryotic microbiota can be evaluated through high-throughput sequencing of nuclear ribosomal ITS1 and ITS2 amplicons and real-time PCR. Results of cloning libraries and ITS1/ITS2 metabarcoding sequencing and real-time PCR revealed the different proportions of intestinal protozoa in stool samples from HIV patients compared to healthy individuals. A more comprehensive view of the eukaryotic population in the intestinal microenvironment of HIV-infected patients could be achieved through the complementarity of different molecular techniques. Combining these various methodologies may provide a more complete characterization of the eukaryotic microbiome in future studies [29,45,71].

In summary, despite their immense and superior advantages over the conventional parasitological methods, molecular approaches face several challenges and limitations. Each method has its individual performance with certain groups of gut eukaryotes. Meticulous consideration of the details in each procedural step will result in better profiling and characterization of the eukaryotic components of the intestinal microbiota [71]. 

## 5. The Future of Intestinal Protozoa Diagnosis

As discussed above, multiplex PCR tests are more sensitive and specific than microscopy for the detection and identification of intestinal protozoa. The Luminex xTAG gastrointestinal pathogen panel has received FDA approval and can simultaneously detect 14 enteropathogens, including *G. duodenalis* and *Cryptosporidium* spp. This test is the first molecular method approved by the FDA for the detection of pathogenic protozoa. Unfortunately, this FDA-approved platform does not include *E. histolytica*, although reagents for these analytes are available for research use only. BioFire Diagnostics is developing a gastrointestinal pathogen panel which includes *Giardia duodenalis*, *Cryptosporidium, E. histolytica*, and *Cyclospora cayentensis*. One of the main criticisms for these multiplex panels is the cost per test, which is much higher than that of the reagent associated with microscopic observation [4]. Moreover, the combination of immunological- and molecular-based methods, such as a nucleotide-based chromatographic test, might allow subtyping of several protozoa at a lower cost.

## 6. Conclusions

Regardless of the extensive research in diagnostic methods, there is no single perfect method to diagnose intestinal protozoa. Each method has varying superiorities, drawbacks, and suitability for different purposes. Methodical deliberation must be performed when selecting the appropriate method or assay depending on the purpose of examination or study, expected parasite, sample type, and availability of resources. Efforts to develop better and optimal detection approaches for intestinal protozoa are continuously performed to explain the unexplored aspects of these eukaryotic members of the intestinal microenvironment.

## Data Availability

Not applicable.
